# Gold Nanoparticles: A New Golden Era in Oncology?

**DOI:** 10.3390/ph13080192

**Published:** 2020-08-12

**Authors:** Clara Gerosa, Guido Crisponi, Valeria Marina Nurchi, Luca Saba, Rosita Cappai, Flaviana Cau, Gavino Faa, Peter Van Eyken, Mario Scartozzi, Giuseppe Floris, Daniela Fanni

**Affiliations:** 1 UOC Anatomia Patologica, AOU Cagliari, University of Cagliari, 09124 Cagliari, Italy; clarge@tiscali.it (C.G.); flacau@tiscali.it (F.C.); gavfaa@unica.it (G.F.); 2 Dipartimento di Scienze della Vita e dell’Ambiente, University of Cagliari, 09042 Cagliari, Italy; nurchi@unica.it (V.M.N.); cappai@unica.it (R.C.); 3 UOC Radiologia, AOU Cagliari, University of Cagliari, 09042 Cagliari, Italy; lucasaba@unica.it; 4 Department of Biology, College of Science and Technology, Temple University, Philadelphia, PA 19122, USA; 5 Department of Pathology, Genk Regional Ziekenhuis, 3600 Genk, Belgium; peter.vaneyken@skynet.be; 6 UOC Oncologia Medica, AOU Cagliari, University of Cagliari, 09042 Cagliari, Italy; marioscartozzi@unica.it; 7 Pathologische Ontleedkunde K.U. Leuven, 3000 Leuven, Belgium; giuseppe.floris@uzleuven.be

**Keywords:** gold, nanoparticles, oncology

## Abstract

In recent years, the spectrum of possible applications of gold in diagnostics and therapeutic approaches in clinical practice has changed significantly, becoming surprisingly broad. Nowadays, gold-based therapeutic agents are used in the therapy of multiple human diseases, ranging from degenerative to infectious diseases and, in particular, to cancer. At the basis of these performances of gold, there is the development of new gold-based nanoparticles, characterized by a promising risk/benefit ratio that favors their introduction in clinical trials. Gold nanoparticles appear as attractive elements in nanomedicine, a branch of modern clinical medicine, which combines high selectivity in targeting tumor cells and low toxicity. Thanks to these peculiar characteristics, gold nanoparticles appear as the starting point for the development of new gold-based therapeutic strategies in oncology. Here, the new gold-based therapeutic agents developed in recent years are described, with particular emphasis on the possible applications in clinical practice as anticancer agents, with the aim that their application will give rise to a new golden age in oncology and a breakthrough in the fight against cancer.

## 1. Introduction

**Figure 1:**
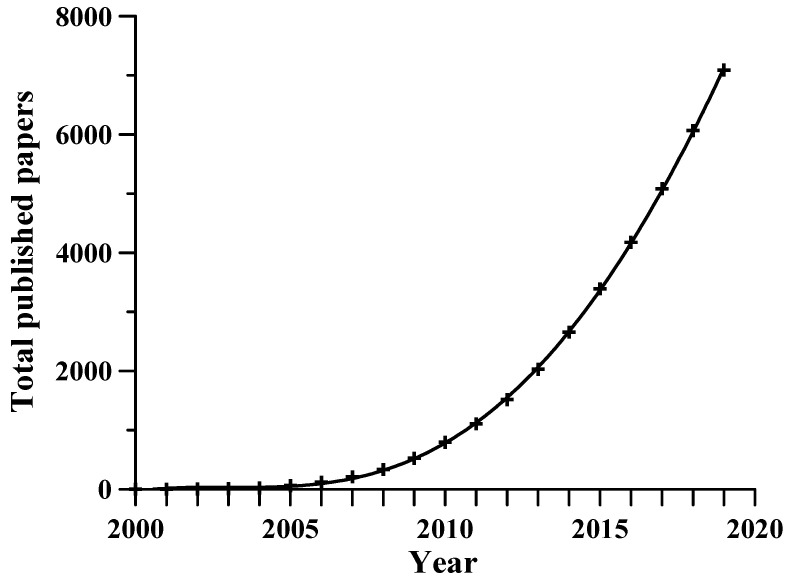
The number of published papers on applications of gold nanoparticles (AuNPs) in oncology in the period 2000–2019. Scopus research using the search terms “gold nanoparticles” and “cancer”.

Research into gold-based drugs for a range of human diseases has seen a revival in recent years, revealing a new potential for an old metal, with the perspective of multiple applications in clinical practice [1]. A great deal of interest in the introduction of gold compounds in oncology has emerged [2,3]. From research on Scopus, using the search words gold nanoparticles and cancer, it was found that the first paper on this topic was published in 1999, and in the first decade of the 2000s (2000–2009), 525 papers were published. In the second decade (2010–2019), 6565 papers were published, a result almost 13 times greater than the previous one. Figure 1, which reports the total publications in the years, highlights the exponential increase of the research in this field.

The introduction in anticancer therapy of gold complexes characterized by relevant antitumor properties has been defined as “the Midas touch” in oncology [4]. The success of gold-based techniques in oncology depends mainly on the ability of gold nanoparticles to selectively accumulate into cancer cells, participating as main actors in the spectrum of target therapy [5]. Gold nanoparticles can absorb X-rays and produce secondary electrons and photons that kill the surrounding malignant cells [6]. Among the available nanoparticles, gold nanoparticles have several advantages: they are biocompatible, can be synthesized in a wide range of sizes, ranging from 1 up to 50 nm, and may be coated with a large number of molecules, including chemotherapy drugs [7].

The aim of this review is the description of the new gold-based therapeutic and diagnostic agents developed in recent years, giving particular emphasis to the possible applications in clinical practice as anticancer agents. Table 1 depicts the main arguments that will be taken into consideration in this work.

## 2. General Considerations of Gold Nanoparticles

The term “nanoparticle” defines a group of atoms or molecules characterized by a diameter between 2 and 100 nm. A nanoparticle can be considered the simplest structure, with a size in the range of nanometers.

**Figure 2:**
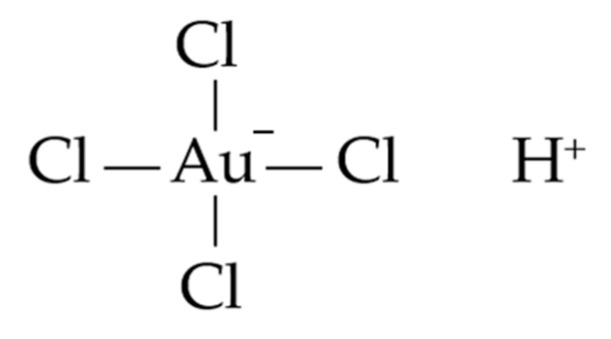
Structure of chloroauric acid.

Gold nanoparticles (AuNPs) are generally produced by reduction of chloroauric acid (Figure 2): the Au^3+^ ions are reduced to neutral gold atoms by means of different reducing agents; when the solution becomes supersaturated, gold precipitates in subnanometer particles. The use of stabilizing agents is necessary to prevent the aggregation of the gold particles. The state-of-the-art in AuNP synthesis has been thoroughly reviewed in the comprehensive work by Zhao et al. [8] and in some recent publications [9,10].

Several varieties of nanoparticles with biomedical relevance have been developed in recent years, including metal nanoparticles, polymeric nanoparticles, liposomes, micelles, quantum dots, and dendrimers. AuNPs may be functionalized by adding different organic ligands that give rise to organic–inorganic hybrids, according to the peculiarities of the target cells. The composition of the coating may be tailored to yield nanoparticles capable of aggregating exclusively when both UV and CO_2_ are applied [11]. Nanoparticles offer unique approaches to studying a variety of biological processes occurring at nanometer scales, both in physiological and pathological conditions. Given the ability of nanoparticles to enter inside cells, nanotechnology is expected to have a revolutionary impact on biology and medicine and to give rise to a new branch of medical sciences, nanomedicine [12]. Among the multiple approaches aimed at introducing nanotechnology in clinical practice, gold nanoparticles offer unique advantages in sensing, image enhancement, and delivery agents. Other remarkable properties of AuNPs are facile synthesis, a stable nature, surface plasmon resonance, surface chemistry, and multifunctionalization [13]

In the following sections, we present some strictly connected aspects that can be considered critical in the drug delivery of AuNPs for cancer treatment.

### 2.1. PEGylation

A delivered drug should circulate in the blood as much time as possible in order to reach the target tissue in a suitable concentration. As drugs are rapidly metabolized and scavenged from the bloodstream, one of the means for prolonging their residence time is to coat the molecules with an inert polymer, usually polyethylene glycol (PEG), which protects the drug from deleterious interaction with the constituents in the bloodstream. In particular, the PEGylation of nanoparticles shields them and extends the circulation time in the blood [14]. PEGylation is advantageous for the reason that the coating of nanoparticles protects them from cellular uptake and adds a hydrophilic moiety to the molecule.

### 2.2. EPR Effect

Matsumura and Maeda reported in 1986 that the blood vessels of the majority of solid tumors are characterized by an enhanced vascular permeability, which allows the accumulation of antitumor agents in the tumor tissue [15]. They named this the EPR effect (enhanced permeability and retention), the mechanism by which large molecules accumulate in tumors. Based on the EPR effect, pharmaceutical nanoparticles with a higher circulation time in the blood are collected in tumor tissues with enhanced vascular permeability, and this tool is commonly used for selective drug delivery into tumors through passive accumulation. In fact, EPR drug delivery does not work on normal tissues [16].

### 2.3. AuNPs and Photothermal Therapy in Oncology

Recent advances in nanomedicine have stressed on the possible introduction of photothermal therapy into clinical practice for cancer treatment by combining nanomedicine and laser. Photothermal therapy employs photothermal agents such as AuNPs, with high photothermal conversion efficacy for converting light into heat, to selectively kill cancer cells with the help of lasers [17]. The low thermotolerance of tumor cells, associated with the high affinity of tumors for AuNPs, is at the basis of the multiple ongoing clinical trials on the efficacy of photothermal therapy in oncology [18]. Tumor cell death is induced when a temperature of at least 43 °C is obtained inside the tumor mass for a period of 15 min. By utilizing pulsed laser ablation, the laser irradiation power and the different concentrations of AuNPs [19]. Coating gold NPs with silica has a shape-conserving effect, preventing gold NPs from deforming following laser irradiation [20]. Regarding the photothermal efficiency of AuNPs, tumor cells containing gold–silica NPs undergo cell death by apoptosis during photothermal treatment. Moreover, the efficiency of gold–silica NPs might be increased by surface silanization with tumor cell ligands specific for each tumor entity, allowing a more specific approach toward different biomedical applications in oncology [19]. The combination of AuNPs-based photothermal therapy with other anticancer therapies, which allows the use of multiple mechanisms that target the growth and survival pathways of tumor cells, might represent a key for improving treatment outcomes in multiple fields of oncology [21].

## 3. Gold Nanoparticles of Different Size, Shape, and Composition

**Scheme 1:**
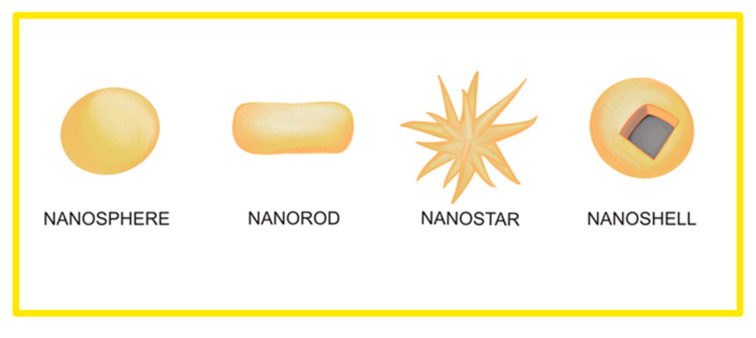
Principal shapes assumed by AuNPs.

Several forms of AuNPs have been developed during the years for therapeutic purposes. They differ in size, wavelength of maximal absorption, and the absorption cross-section. The selection of the AuNP variants to use in a particular pathological setting represents the first and most important decision when choosing a gold nanoparticle [22]. Scheme 1 presents the principal shapes assumed by AuNPs.

### 3.1. Colloidal Gold Nanospheres

Artists have made use of colloidal gold nanoparticles for centuries for the exciting colors caused by the interaction of nanoparticles with visible light. The origin of these colors is due to the occurrence of the so-called “localized surface plasmon resonance” (LSPR), i.e., the conduction electrons on the surface of a nanoparticle oscillate in resonance with incident visible radiation. LSPR can be modified by changing the size or the shape of the nanoparticles, and this gives the opportunity to obtain particles with optical properties tailored according to the different possible applications. Michael Faraday was the first, in 1857, to obtain colloidal AuNPs by reacting gold chloride with sodium citrate [23]. The nanoparticles that work the best for targeting tumor cells are 35 nanometers in size [24].

### 3.2. Gold Nanorods

Gold nanorods are characterized by small size, ranging from 10 up to 50 nm, and by high absorption coefficients. As compared to near-infrared (NIR)-absorbing AuNPs, gold nanorods offer a higher photothermal heating efficiency. In experimental models carried out on nude mice, inhibition of tumor growth and dramatic size decrease were observed in squamous cell carcinoma xenografts by photothermal therapy following direct and intravenous administration of PEGylated gold nanorods [25]. Recent studies have confirmed that gold nanorod-assisted plasmonic photothermal therapy may be considered a promising approach for new anticancer strategies, thanks to the ability of gold nanorods to absorb NIR radiation and to convert it into heat, causing tumor cell death by apoptosis and/or necrosis. A 15-month toxicity study in mice xenografts showed no long-term toxicity of gold nanorods in vivo, providing a strong framework for the translation of gold nanorod-based photothermal therapy to clinical practice [26].

### 3.3. Gold–Silica Nanoshells

They are composed of silica cores covered with gold. The resonance of gold–silica nanoparticles spans from the infrared to the visible. Moreover, gold–silica nanoshells have been the first to show photothermal activity by inducing cancer cell death, converting light into heat [17,18]. Recently, a novel nanoplatform based on silica nanoparticles was constructed, aimed at developing chemo/photothermal therapy to enhance gold nanoparticle accumulation inside tumor cells and increasing the toxicity of chemotherapeutic drugs [27].

### 3.4. Small NIR–Tunable Gold Nanoparticles

Gold sulfide nanoparticles are smaller than gold–silica nanoshells, with a diameter down to 25 nm, and have a NIR absorbance that may be utilized for thermoablative anticancer therapy. Their potential in oncology has been demonstrated in xenografts of human prostate cancer in mice [28].

### 3.5. Hybrid Gold–Albumin Nanoparticles

The anticancer efficiency of AuNPs and their possible introduction in clinical practice have been halted by their poor in-vivo stability and by their potential toxicity. In order to increase the affinity of AuNPs for tumor cells, metallic nanoclusters have been associated with plasma proteins, with the aim of increasing their efficacy in the imaging and therapy of cancer [29]. Small gold nanorods were encapsulated with albumin, with the aim of utilizing biocompatible albumin as a carrier. Hybrid gold–albumin nanoparticles showed higher tumor targetability and photothermal activity [30]. According to this study, hybrid gold–albumin nanoparticles should be considered promising photothermal agents, with excellent tumor ablation, good targetability, and lower toxicity, compared to nanoparticles alone.

### 3.6. Gold Nanorod–Encapsulated Biodegradable Polymeric Matrix

A biocompatible nanocomplex system of polyethylene glycol and dodecane was coencapsulated with gold nanorods and doxorubicin (DOX) [31]. The anticancer effects of the encapsulated nanorods were analyzed on various cancer cell lines and in xenografts in nude mice. A strong photothermal effect on tumor cells was verified, associated with the rapid release of the chemotherapy drug doxorubicin. DOX treatment, joint to ionizing radiation, also increases the number of double-strand breaks of DNA [32]. Radiotherapy, using AuNPs to deliver DOX into cancer cells, has also been taken into consideration [33], and the resulting enhancement in cell killing was ascribed to the synergistic action of DOX cytotoxicity and AuNP radiosensitization [34,35].

### 3.7. PEGylated AuNPs

Another problem related to the efficacy of AuNPs as anticancer agents is their recognition by the immune system, ending with their phagocytosis by the cells of the reticuloendothelial system and clearance from the bloodstream [13,36]. As explained in Section 2.1, to escape fast immune recognition and clearance, AuNPs have been coated with a polyethylene glycol (PEG) layer by a process defined as “PEGylation”, which masks the AuNP surface and prevents their recognition by the immune system, prolonging their persistence and activity [37].

### 3.8. Paramagnetic AuNPs

Hybrid nanoparticles, made of a gold shell covered with supermagnetic iron oxide, have been developed to be utilized as a dual contrast agent for computed tomography (CT) and magnetic resonance (MR) imaging, due to the high attenuation of CT and good MR signals [38].

## 4. Gold Nanoparticles in Oncology

### 4.1. AuNPs in Cancer Imaging and Detection

Noble metal nanoparticles, including AuNPs, are characterized by their strong surface fields that are at the basis of the high absorption and scattering of electromagnetic radiation. Moreover, AuNPs may alter cellular auto fluorescence of NADH and collagen, suggesting their use as optical probes for the fluorescence-based detection of cancer cells [39]. AuNPs concentrated into tumor cells absorb much light than would normally be expected, whereas the light that is not absorbed is strongly scattered. Due to their strong scattering, AuNPs show the potential for cancer imaging in the early diagnosis of tumors. Ultrasmall gold nanoparticles have been proposed, in recent years, for both cancer diagnosis and treatment. Multiple methods have been developed for controlling the size and surface of ultrasmall gold nanoparticles for their use in cancer imaging and treatment. The applications of ultrasmall AuNPs in tumor visualization and bioimaging have been proposed in different fields of radiology, including magnetic resonance imaging, photoacoustic imaging, fluorescence imaging, and X-ray scatter imaging [40].

### 4.2. AuNPs in Anticancer Therapy

**Figure 3:**
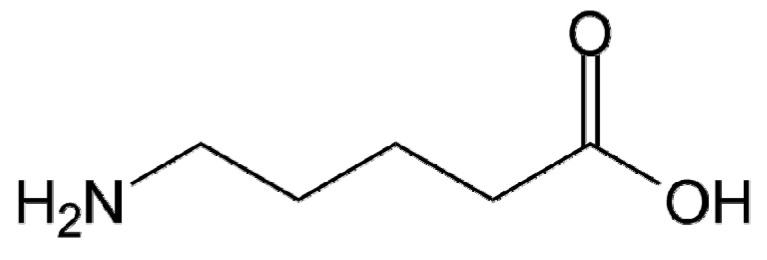
Structure of 5-aminovaleric acid.

In recent years, the metallic system, and in particular gold, has become more and more attractive for cancer therapy. Studies carried out on experimental models have shown that mice injected with cancer cells and AuNPs, when submitted to X-ray therapy, were characterized by tumor reduction or eradication. On the contrary, in mice treated only with X-ray or with gold alone, the growth of tumor cells was not halted, suggesting a synergistic effect between gold and radiotherapy [41,42]. In short, AuNPs might play a key role in improving radiotherapy efficiency as radiosensitizers [43]. An improvement in the use of AuNPs for therapeutic applications was obtained by covering gold with organic compounds such as amino acids and amino sugars, which act as vehicles that transport the nanoparticles into the tumor cells [44,45]. The stabilization of AuNPs close to 7 nm in diameter with 5-aminovaleric acid (Figure 3) allows the nanoparticles to selectively penetrate into human leukemia cancer cells in culture after just 15 min of contact [5]. Colloidal gold-based nanoparticles have been designed to target the delivery of tumor necrosis factor (TNF) and paclitaxel to solid tumors, introducing AuNPs as tumor-targeted drug delivery vectors [46]. In a clinical trial approved by the FDA, whose first phase has been completed, novel PEGylated AuNPs were utilized to deliver TNF into cancer cells, ending with selective TNF storage in tumor cells [47].

AuNPs synthesized utilizing tetrachloroauric acid as the gold source and sodium tetrahydroborate as the reductant have been utilized for enhancing radiation therapy sensitivity in radiation-resistant human prostate carcinoma cells in vitro.

**Figure 4:**
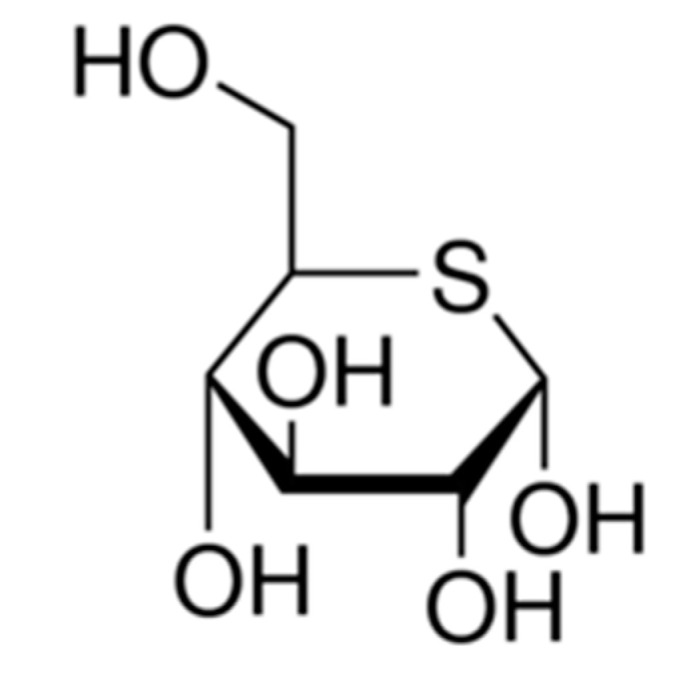
Structure of 5-thio-D-glucose.

The addition of thio-glucose (Figure 4) to the solution allows us to obtain thio-glucose-capped gold nanoparticles, whose uptake by tumor target cells was three times higher than nude AuNPs. Glucose-bound AuNPs enhanced radiation sensitivity, ending with growth inhibition of tumor cells in prostate cancer [48]. These data suggest that radiation therapy combined with AuNPs might represent a new efficient therapeutic approach in anticancer therapy. Of some relevance is the ability of glucose coating to increase the uptake of AuNPs by cancer cells by improving the radiosensitivity of tumor cells.

AuNPs are considered excellent drug and anticancer carriers, with many biochemical and therapy applications [49]. An important peculiarity of AuNPs is their high affinity for tumor cells, about 600 times greater than for surrounding nontumor cells [24]. A further benefit of AuNPs is represented by their very fast uptake by cancer cells [50]. In ovarian cancer cells in culture, AuNPs significantly affected cell proliferation activity within 12 h from the treatment [51]. No clear conclusion regarding the intimate mechanism by which gold nanoparticles induced radiosensitization in tumor cells has been reached. Tissue hypoxia has been hypothesized to play a major role in their uptake, which is higher under hypoxic than aerobic conditions [52]. A recent study on the mechanisms underlying radiosensitization exerted by gold nanoparticles remarked their activity in the three phases of activity of the ionizing radiations:

(i) the physical phase, in which free radicals are produced; DNA being the main target of the cascade of ionization events.
(ii) the chemical phase, in which highly reactive radicals fix the damage.
(iii) the biological phase, in which cellular repair processes are activated to repair the damage or, alternatively, to trigger the apoptotic cascade, ending with cell death [35].

As far as their entry inside the cells is concerned, AuNPs are mainly localized in the cytoplasm of target cells, where they increase the expression of endoplasmic reticulum stress-related proteins, inhibit the expression of DNA repair-related proteins, and promote apoptosis, thus increasing the efficacy of tumor-target chemotherapy [53].

The delivery of chemotherapy agents using gold nanoparticle–drug conjugates in combination with radiotherapy is at the basis of a new field in oncology: the simultaneous chemo–radiotherapy. This new therapeutic approach may represent an important tool in anticancer research that is able to improve antitumor treatment outcomes in chemo-resistant and radio-resistant tumors [54]. Gold nanoparticles with a size of ∼13 nm have been shown to concurrently possess superior CT contrast ability and significant radioactive disruption, allowing both enhanced CT imaging and radiotherapy [55].

### 4.3. AuNPs as Photothermal Therapeutic Agents

Lighting gold nanoparticles with IR laser radiation after their uptake by tumor cells represents a promising approach that might be utilized in clinical practice and, in particular, in oncology, both for diagnostic and therapeutic purposes [21,56,57]. With the development of gold nanoparticles designed for photothermal therapy applications and of miniaturized light-delivery systems, phototherapy will be able to access deep tumors located at anatomical sites in our body that are hard to reach with classical therapeutic approaches [21]. These data taken together, sustain the hypothesis that gold nanoparticle-mediated hyperthermia might represent a new era for photothermal therapy and, in particular, for anticancer therapy [22]. Modifying the shape and size of gold nanoparticles can lead to a modification of the photochemical activity. In fact, the variation of photothermal features permits the use of radiations of different wavelengths, such as NsIR, and this can open new directions for the application of AuNPs in cancer treatment [58,59]. Recently, the combination of gold nanoparticles-based photothermal therapy and chemotherapy has been utilized to achieve synergistic anticancer effects, giving rise to a new branch of oncology defined as chemo–photothermal therapy (CPTT) [60]. Nanotechnology, and in particular gold nanoparticles, are emerging as a promising strategy to enhance radiotherapy efficacy in multiple fields of oncology, both for diagnostic and therapeutic approaches. The preferential accumulation of gold nanoparticles inside tumor cells leads to (i) tumor-specific delivery of antitumor chemotherapeutic agents for combined chemo–radiotherapy, (ii) increased local dose of radiation, due to the high X-ray absorption coefficient of gold, and (iii) improved contrast enhancement for image-guided radiotherapy [35].

## 5. AuNPs in Specific Tumor Entities

### 5.1. Pancreatic Cancer

**Figure 5:**
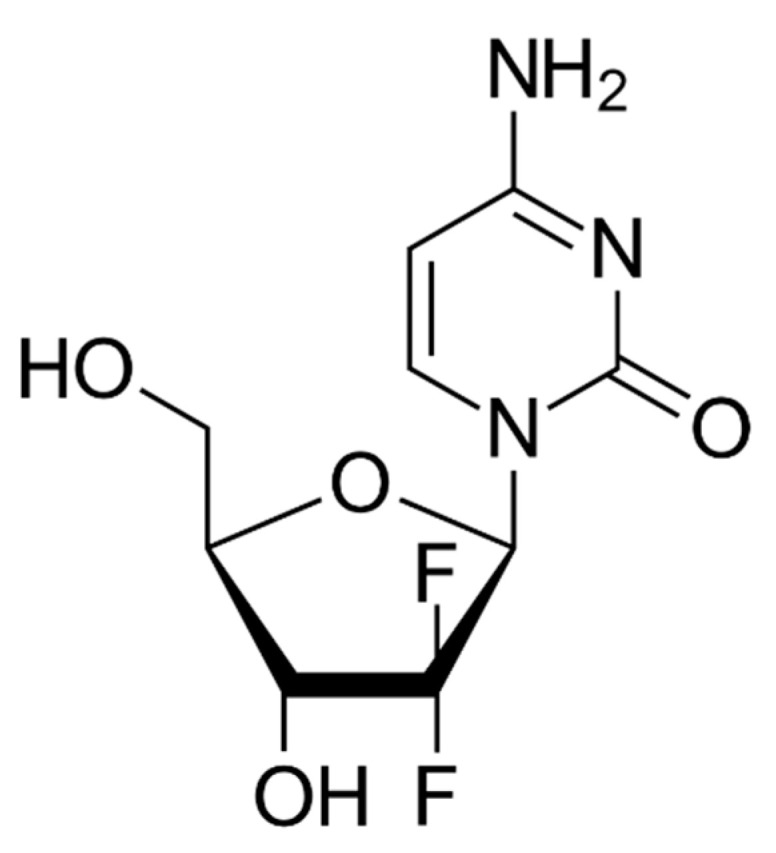
Chemical structure of gemcitabine.

Ductal adenocarcinoma of the pancreas is one of the deadliest solid malignant tumors, with a dismal prognosis. A recent study on the utility of AuNPs in the therapy of pancreatic adenocarcinoma showed that AuNPs of 20 nm had a relevant effect on pancreatic cells by inhibiting their capacity of migration and colony-forming. Furthermore, pancreatic cells were sensitized to gemcitabine (Figure 5) in the assays of viability and colony formation [61].

AuNPs can disrupt growth factor-mediated signaling and reverse epithelial mesenchymal transition (EMT), leading to the inhibition of tumor growth in pancreatic cancer cells [62]. In the same study, AuNPs inhibited pancreatic tumor cell growth by decreasing the expression of angiogenetic factors, including vascular endothelial growth factor (VEGF), epithelial growth factor (EGF), and fibroblast growth factor (FGF). Pretreatment with AuNPs halts the EMT process and reverses the translocation of E-cadherin from the membrane to the cytosol back to the membrane, favoring the adhesion of pancreatic tumor cells among them and contrasting their detachment and migration. 

Cancer-associated fibroblasts (CAFs) are the third element that might be influenced by AuNPs in pancreatic cancer. Gold nanoparticles inhibit the proliferation and migration of CAFs, thereby preventing the crosstalk between the pancreatic cancer cells and the peritumoral fibroblasts. This ability of AuNPs is probably related to their activity in modulating the cellular secretome to reduce the growth of desmoplastic tissue and inhibit tumor cell growth [63].

These results suggest that gold nanoparticles sensitize pancreatic cancer cells to gemcitabine. Moreover, gold nanoparticles have shown a previously unknown potential to act as stand-alone therapeutics and should be considered as a potential new therapeutic tool, not only to sensitize pancreatic cancer cells to gemcitabine but also as therapeutic agents alone [64].

Gold nanoparticle-incorporated molecularly imprinted polymer microgels (Au–MIP microgels) have been recently proposed as radiation sensitizers. In particular, the effects of radiation sensitization were studied on mice with pancreatic cancer by injection of Au–MIP microgels.

The tumor size in mice injected with the Au–MIP microgels was smaller than that in control mice, indicating that Au–MIP microgels might have applications as novel radiation sensitizers in antipancreatic cancer radiation therapy [65].

### 5.2. Colon Cancer

The efficacy of AuNP-based photothermal therapy in colon cancer cells has been evaluated in a murine subcutaneous colon cancer model. In this study, first, gold nanorods were infused, and then xenografts underwent percutaneous illumination with an 808-nm laser [66]. The survival of the gold–photo thermally treated mice was statistically longer than that of control animals, confirming that gold nanorods represent a new promising agent for photothermal ablation. According to these findings, nanogold-based photothermal therapy should gain great attention in colon cancer therapy. Recently, the multiple pathways adjuvanted by AuNPs in colon cancer cells, leading to the apoptosis of tumor cells, have been elucidated. AuNPs enhance reactive oxygen species (ROS) generation, damage mitochondrial membrane, induce G0/G1 phase cell-cycle arrest, and activate caspase expression, ending with apoptosis cell death [67]. An in-vitro study on gold nanoparticles obtained by green synthesis using *Trichosanthes kirilowii* extracts gave evidence that gold nanoparticles, 50 nm in size, show effective, selective, and anticarcinogenic effects on HCT-116 cells in a dose-dependent manner. The gold nanoparticles significantly enhanced ROS generation, caused mitochondrial damage, and induced G0/G1 phase cell-cycle arrest. Moreover, gold nanoparticle treatment activated caspase expression, inducing apoptosis of tumor cells and showing a highly efficient potential for cancer treatment [67].

### 5.3. Squamous Cell Carcinoma of the Hypopharynx

A recent study carried out on nude mice aimed to investigate the effects of photothermal therapy with gold nanorods on squamous cell carcinoma of the hypopharynx revealed that gold nanorods associated with NIR radiation inhibited tumor growth. Moreover, when gold nanorods were conjugated with monoclonal antibodies against epidermal growth factor receptor (EGFR), the inhibitory effects on tumor growth were enhanced, increasing the antitumor effects of AuNP-induced photothermal therapy [68]. A recent study has proposed gold nanoparticles as an important tool for the early detection of squamous cell carcinoma insurgence in the oral cavity. This novel imaging instrument can lead to significant improvements in oral cancer therapy due to earlier detection, accurate staging, and, above all, the ability to identify micro tumors [69].

### 5.4. Prostate Cancer

**Figure 6:**
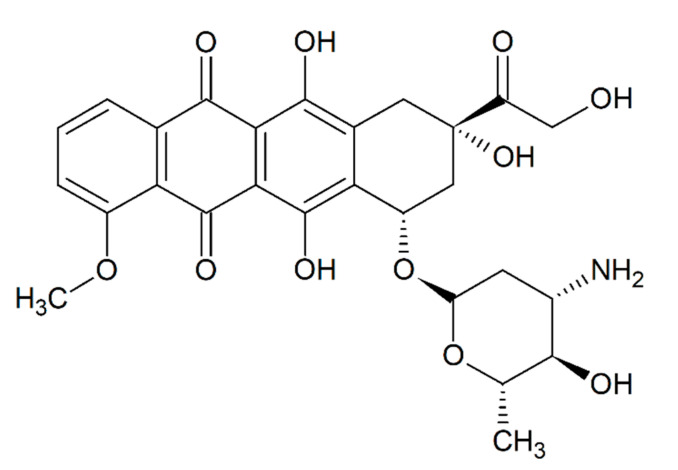
Chemical structure of epirubicin.

In order to analyze the effects of AuNPs on human prostate cancer xenograft in mice, epirubicin, an anthracycline drug (Figure 6), was loaded onto the surface of gold nanorods.

The administration of the complex epirubicin–gold nanoparticles amplified the antitumor activity of epirubicin, ending with a marked antiproliferative activity in prostate tumor cells. Moreover, when the tumor loaded with the complex epirubicin–nanorods was submitted to laser irradiation, the antiproliferative activity was significantly increased [60]. A recent study aimed at developing gold nanoparticles targeted with a prostate-specific membrane antigen, which might significantly improve X-ray therapy, gave evidence of the radiosensitizing activity of AuNPs that allow optimized radiotherapy of prostate carcinoma [70].

### 5.5. Breast Cancer

**Figure 7:**
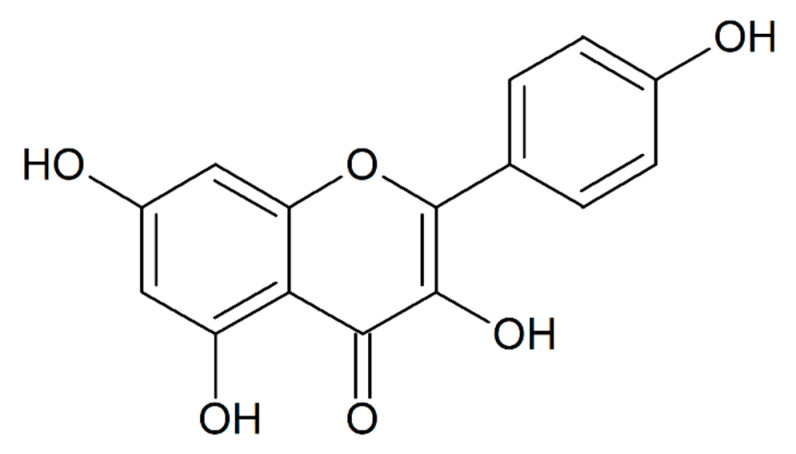
Chemical structure of kaempferol.

**Figure 8:**
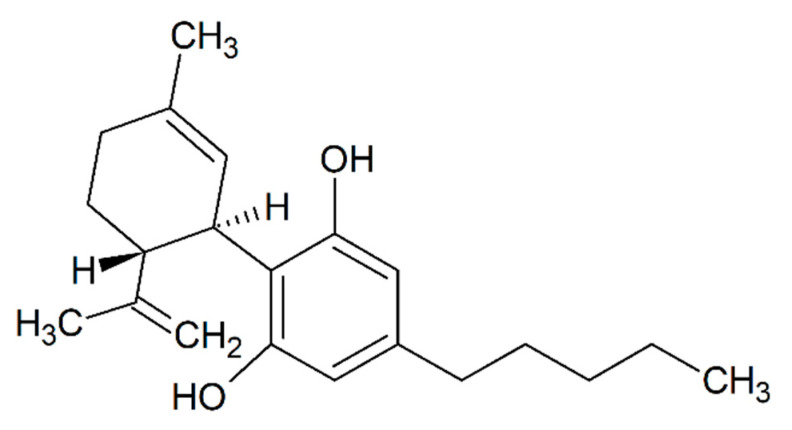
Chemical structure of cannabidiol.

In vitro, gold nanoparticles have been demonstrated to induce apoptosis in human breast cancer cells [71]. By conjugating AuNPs with kaempferol, a phytochemical (Figure 7), their application on breast cancer cells caused a higher cytotoxic effect, demonstrated by high levels of apoptosis, compared to kaempferol alone [72]. Additional studies carried out on a breast cancer model in mice showed that gold nanoparticles, whose size of the gold core was 1.9 nm, were able to efficiently carry the anticancer drugs into tumor cells, increasing the efficacy of radiation therapy, with a marked reduction of tumor volume [73]. These findings suggest that AuNPs could be utilized in the future instead of chemotherapy in the treatment of cancer diseases. A new approach for the treatment of breast cancer was recently proposed, utilizing gold nanoparticle-based photodynamic therapy conjugated with cannabidiol, a derivative from *Cannabis sativa* (Figure 8) [74].

### 5.6. Lung Cancer

**Figure 9:**
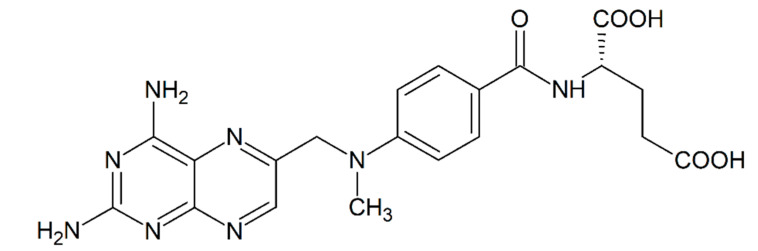
Chemical structure of methotrexate.

AuNPs stabilized with apigenin, a bioactive agent with anticancer functions, were able to increase the efficiency of radiation therapy in cells of human lung carcinoma in culture [75]. This experiment lays stress on the possibility of integrating two therapies, chemotherapy and gold-assisted radiation therapy, for lung cancer treatment. In previous studies, AuNPs conjugated with methotrexate (Figure 9), a chemotherapy agent widely used in oncology, displayed higher cytotoxicity in a lung tumor model [76].

**Figure 10:**
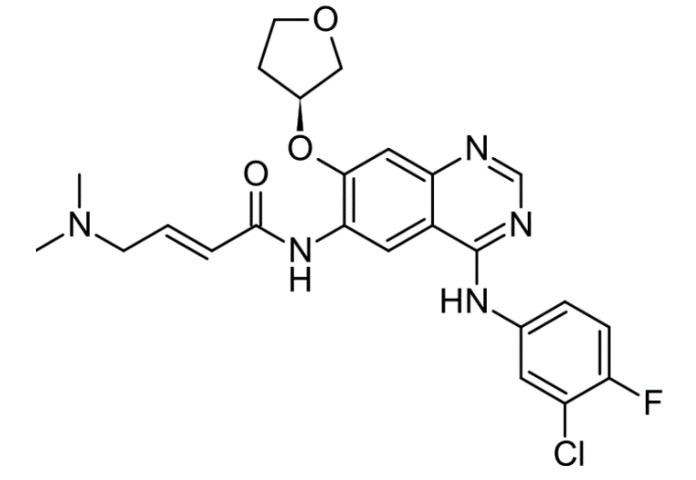
Chemical structure of afatinib.

The development of new AuNPs with betacyclodestrin evidenced their ability to increase the efficiency of radiation therapy in human cancer lung cells [77]. New ecofriendly methods for the synthesis of nanoparticles by using plant extracts have been proposed, substituting the conventional methods. Gold nanoparticles were prepared from *Magnolia officinalis*, identified as an ecofriendly and less toxic method. Leaf extracts have shown potential in reducing chloroaurate ions to form gold nanoparticles, with the advantage of rapid formation of stable AuNPs. The reaction temperature has a great impact on the size of the formed nanoparticles. The anticancer efficacy of these new AuNPs was assessed in A549 lung cancer cells, causing cytotoxicity and tumor cell death by inducing the expression of multiple proapoptotic genes [78]. Chemotherapeutic drugs such as afatinib (Figure 10), used in the treatment of nonsmall cell lung carcinoma (NSCLC), being hydrophobic, have low bioavailability, spread around the body, and cause severe side effects. A novel afatinib–gold nanoparticle formulation termed Afb–AuNPs has been recently developed, with the aim of improving the efficacy and biocompatibility of the drug. These new Afb-conjugated gold nanoparticles were found to possess up to 3.7-fold increased potency when administered to lung cancer cells in vitro and were capable of significantly inhibiting cancer cell proliferation [79].

### 5.7. Hepatocellular Carcinoma

**Figure 11:**
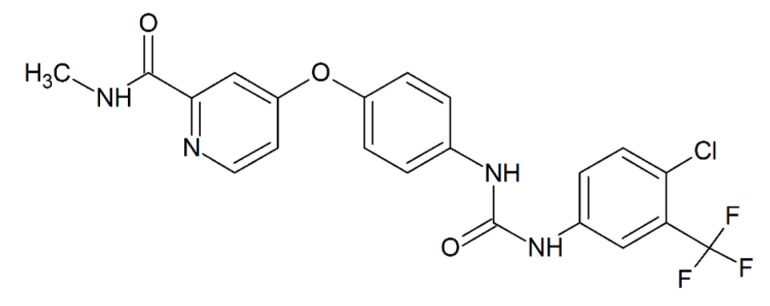
Chemical structure of sorafenib.

Traditional chemotherapy has been widely used for the treatment of hepatocellular carcinoma (HCC) even though the results were not often efficient, due to the poor cellular uptake of chemotherapy drugs and drug resistance. To address this inability to halt cell growth in HCC, a new type of AuNPs was developed, formed by nanoshells for photothermal conversion associated with sorafenib (Figure 11), a first-line anti-HCC chemotherapy drug [27]. The sorafenib–AuNPs accumulated more in hepatocarcinoma tumor cells as compared to sorafenib alone. Moreover, under NIR irradiation, the new gold–sorafenib nanoparticles exerted a high cell inhibition rate, which could be attributed to the enhanced toxicity of sorafenib under hyperthermia. Overall, sorafenib–AuNPs might be a promising candidate for enhancing the antitumor therapy of HCC. Another study showed that sorafenib combined with gold nanoparticles–anti-miR221 synergistically inhibited the proliferation of HCC cell lines [80]. Moreover, in clinical practice, gold nanoparticles have been introduced in different fields of the diagnosis and therapy of liver cancer, including imaging, drug and gene delivery, radiotherapy, and photothermal therapy [81].

## 6. Toxicity of AuNPs

The introduction into clinical practice of gold nanorods is restricted, and the FDA, to date, has approved only a few clinical trials [82]. This is mainly due to their high toxicity for cells and tissues, with unintended side effects on human health. This toxicity is mainly related to the accumulation of AuNPs inside the body [83,84]. For example, AuNPs stored in the liver can cause severe injuries as there is an increase of Kupffer cells, inflammation, and tissue apoptosis [85,86,87]. To elude these problems, AuNPs have to be adequately small, since AuNPs <8 nm, which pass through glomerular filtration, are well excreted from the body without massive accumulation in body tissues [88,89,90]. Numerous methods have been developed for decreasing toxicity, including coating gold nanorods with biocompatible materials. Among them, organic polymers are intensively investigated due to their low toxicity and easy access to modification with functional groups, such as targeting molecules on the tumor cell surface, and imaging agents, such as radioactive elements. According to these data, the detoxification and functionalization of gold nanorods with organic polymers might allow their introduction in clinical practice and their application in cancer photothermal therapy [91]. The size of the gold nanoparticles represents the main factor determining toxicity. In particular, nanoparticles with a diameter <2.0 nm show high toxicity, due to their ability to cross the nuclear pores and enter into the nucleus [92]. Gold nanoparticles with a size larger than 10 nm are characterized by lower cytotoxicity. Moreover, the cytotoxicity of gold nanoparticles has been shown to also related to the different cell types when testing AuNPs via radioactive analyses in multiple cell lines in vitro [93]. Physical properties of AuNPs, including their shape and dispersion state, may also influence their toxicity. Regarding the shape, gold nanospheres and nanorods are more toxic than star, flower, and prism gold nanostructures, probably due to their small size and ability to not damage the cell membranes [94]. Bayal et al. have recently shown that the cytotoxicity of gold nanoparticles is negatively correlated with the viscosity of the cell culture media [95]. In a study aimed at verifying the cytotoxicity of gold nanorods (≈39 nm length, 18 nm width), gold nanostars (≈ 215 nm), and gold nanospheres (≈ 6.3 nm) against osteosarcoma and pancreatic carcinoma cell lines, gold nanostars showed the highest anticancer potential. They were the most cytotoxic among the tested nanoparticles, confirming the major role of nanoparticle shapes in their anticancer potential [96]. Another factor regarding the uptake and toxicity of gold nanoparticles has been identified, i.e., the influence of serum proteins in forming different levels of biological corona on gold nanoparticles, thereby influencing the nano–bio interface. Increased uptake levels were described for gold nanospheres stabilized with amino acids, compared to citrate or cetyltrimethylammonium bromide. When measuring cytotoxicity, rod- and cube-shaped particles were well tolerated by the cells, whereas higher toxicity levels were detected for spherical and prismatic particles [97].

## 7. Concluding Remarks: The Future of the AuNPs in Oncology

The new research field of nanobiotechnology has been undergoing rapid development over the past decade. Recent advances in nanotechnology are showing that targeted radiation therapy (RT) with gold nanoparticles should be considered an important mainstay in oncology, by increasing the ability of RT to specifically target cancer cells and further increase the RT therapeutic ratio [98]. In fact, a high number of AuNPs with different sizes and shapes have been synthesized and are under investigation for their properties and biomedical behavior [99]. For biological applications and, in particular, for the introduction of AuNPs into clinical practice in oncology, the production of gold nanoparticles with long-time stability is an indispensable requirement. To this end, new strategies are emerging for the large-scale production of AuNPs. Among these, pulse laser ablation in liquid (PLAL) probably represents the future of NP synthesis, being able to produce NPs in solution, increasing the stability and integrity of gold nanoparticles [19]. The improvements in the future, the possibility of obtaining AuNPs with a wide range of shapes and sizes, from spherical to bone-shaped, with different physicochemical properties, which will allow their application in multiple fields of oncology [10]. In the near future, gold nanoparticles are expected to play a major role in the therapy of a large number of tumors, with the hope of revolutionizing current methods and strategies for cancer treatment [59].

## Figures and Tables

**Table 1:** Comprehensive table of the treated topics.

Section	Title
Section 2.	General considerations on gold nanoparticles
Section 3.	Gold nanoparticles of different size, shape, and composition
Section 4.	Gold nanoparticles in oncology
Section 4.1.	AuNPs in cancer imaging and detection
Section 4.2.	AuNPs in anticancer therapy
Section 4.3.	AuNPs as photothermal therapeutic agents
Section 5.	AuNPs in specific tumor entities:
Section 5.1.	Pancreatic cancer
Section 5.2.	Colon cancer
Section 5.3.	Squamous cell carcinoma
Section 5.4.	Prostate cancer
Section 5.5.	Breast cancer
Section 5.6.	Lung cancer
Section 5.7.	Hepatocellular carcinoma
Section 6.	Toxicity of AuNPs
